# Recent Publications on Anatomy

**Published:** 1838-01-01

**Authors:** 


### SOME RECENT PUBLICATIONS ON ANATOMY.

I. OBSERVATIONS ON THE STRUCTURE AND FUNCTIONS OF THE SPINAL CORD. By *R. D. Grainger*, Lecturer on Anatomy and Physiology. 8vo., pp. 159. London, 1837.

II. and III. GUY'S HOSPITAL REPORTS, Nos. IV. and V. October, 1837.

IV. ELEMENTS OF PHYSIOLOGY. By *J. Müller*, M.D., Professor of Anatomy and Physiology in the University of Berlin, &c. Translated from the German, with Notes, by *William Baly*, Member of the Royal College of Surgeons, &c. Illustrated with Steel Plates and numerous Wood Engravings. Part I. Containing General Physiology; the Blood and Circulating System; the Lymph and Lymphatic System; Nutrition, Growth, and Reproduction. 8vo., sewed, pp. 428. London, 1837. Price Nine Shillings.

V. ELEMENTS OF ANATOMY. By *Jones Quain*, M.D. Fourth Edition, revised and enlarged. Illustrated with Steel Plates and numerous Engravings on Wood. Part I. Price Twelve Shillings. 8vo., sewed, pp. 491. London, 1837.

VI. ANATOMICAL PLATES. By *Jones Quain*, M.D.

IT would be idle in us insisting on the importance of anatomical study. When medicine was more empirical and less scientific than it now is, when its anatomical basis was little understood, then the anatomist was looked on as a sort of scholiast, neither adapted for, nor conversant with, the practical offices of the profession. But so soon as surgery became a science, and the investigation of the tissues gave the breadth of generalization to the previous observations of the empirical physicians, it began to be less and less an axiom, that attention to anatomy was a source of disqualification for practice.

At the present day, it will be found that the best physicians and surgeons have either been engaged in teaching anatomy, or have paid considerable attention to it. The old race of empirical routinists is fast dying away; and the young men who are rising into notoriety are honourably distinguished by their sound scientific education.

Anatomy, however, is an eminently progressive science. Being one of facts, it is capable of accumulative advancement, for a single unit added to any number of units makes that one the more. But it is not simply from the addition of a few fresh facts that Human Anatomy is progressive; it is from the application of the analogical method to it. Comparative anatomy, and, consequently, comparative physiology, remained nearly stationary from the time of Aristotle down to that of Daubenton. Cuvier generalized, Hunter reasoned, and Blumenbach, and the St. Hilaires, and Carus, and a host of others, dissected, compared, and systematized. The results of their labours are now beginning to appear. General physiology is rearing its head as one of the most noble, most philosophical, and most delightful branches of human knowledge; and, while it is highly beautiful in itself, it is doing for human physiology that which general anatomy had already done for human anatomy—it is merging special facts into general laws, and shewing that even what appeared exceptions are fragments of general laws themselves.

It is impossible for one who is not always in the schools to keep pace with the advance of elementary knowledge, unless he is assisted by the periodical press. That carries to him in a cheap, a condensed, and generally in an engaging form, the new facts and new doctrines daily bubbling up through the busy and fermenting mass of intelligence. A well-informed editor of a journal of science is both an useful and an honourable member of the profession to which he may happen to belong. If his own mind has a bent towards true philosophy, he must necessarily promote, in a sensible degree, the general taste for it. And perhaps there are few reflections more delightful than the consciousness on the part of any man, that, by his humble efforts, he is assisting the great cause of truth, and advancing the real welfare of his species.

We have lately adopted the plan, which we believe to be a good one, of directing the attention of our readers into particular channels. It is folly to suppose that the mass of medical men can be made bibliographers, or transcendentalists, or Scriblerus's. They must attend to the every-day duties of an arduous profession, exhausting their physical, and painfully engaging their mental powers. To such men periodicals are eminently necessary, and the kind of information they should bring is such as will apply more or less directly to the practical business of their lives. The leading facts, the great generalizations, a sketch of the advance of knowledge, and, above all, a right direction of their minds, are the things they want, the things which journals should give.

It is in accordance with such views that we have insisted so continually on the cultivation of an exact spirit of enquiry—that we have dwelt on the importance of morbid anatomy—that we have urged the application of statistics—that we are prompting hospital surgeons and physicians to the publication of clinical reports—that we have directed attention to the advances of anatomy. Our aims, in short, and we have frequently proclaimed them, are utilitarian in the highest and the widest sense. But we have said enough.

The first work to which we shall advert is that of Mr. Grainger.

1. ON THE STRUCTURE AND FUNCTIONS OF THE SPINAL CORD.

The objects of Mr. Grainger can hardly be explained so well as in the words of his own preface. And that explanation is necessary to the reader, who wishes to comprehend the objects of the author.

" In the present treatise," he says, " an attempt is made to obtain some definite information, respecting a subject of the highest physiological import,—the true seat of sensation. A reference to the established doctrines is sufficient to convince every person whose judgment is not biassed by theories, as fallacious as they are universal, that our knowledge of the properties possessed by the nervous system, is not only inadequate satisfactorily to elucidate any one function of the animal economy ; but is, in more than one respect, *absolutely opposed to the dictates of common sense.* In contemplating the operations of the inorganic world, nothing is perceived but harmony, regularity, and exactness; whilst, if we regard the phenomena of nature, in the animal and vegetable kingdoms, as they are now interpreted, we discern only confusion and uncertainty.

The total insufficiency of the principles of physiology, as they are at present taught, is universally acknowledged ; and a strong and daily increasing conviction has arisen, that the time is not far distant, when the scattered facts with which this science abounds, will be shown to depend on a few simple and general laws. The magnificent discoveries of comparative and developmental anatomy, by demonstrating the wonderful uniformity which prevails in the construction of animal bodies, plainly evince that such an anticipation is not visionary ; for it would be an unparalleled anomaly in the laws of creation, if such unity of organization as is displayed, not only in the nervous, osseous, glandular, and other systems, but in the formation of the entire frame, were not accompanied by a corresponding simplicity in the laws which regulate the actions of this perfect machinery.

An extended and careful examination of the reflex power of the spinal cord, discovered by Dr. Marshal Hall and Professor Müller, has induced me to believe that it is only a part of a great principle, connected with the nervous system ; from the application of which, in the investigation of all those motions which have their source in contractility, the most valuable results may be anticipated, both in the animal and vegetable kingdoms. The laws by which this important power is regulated, are as simple and exact as those of gravity itself; and it is this circumstance, more especially, which seems to indicate the existence of an universal principle in the movements of organised bodies.

One of my principal objects has been to detect the anatomy by which the reflex power operates ; and, although this branch of the inquiry needs much further prosecution, I am, myself, convinced that a peculiar order of nerves, called the excitomotory, not only exist in the cerebro-spinal, but, likewise, in the ganglionic system. The contractile power possessed by plants, has induced many anatomists to conclude that those bodies are provided with some kind of nervous system ; and, if it should be proved, hereafter, that the vessels and tubes of vegetables do act in obedience to the reflex principle, it is certain that they must be furnished with organs which, however much they may be modified in their physical characters, correspond in office with the excito-motory system of animals.

It may, perhaps, be thought, that more importance is attached to the anatomy connected with this principle than it deserves ; but although no one is more willing than myself to acknowledge the profound spirit of physiology, which enabled Dr. Hall, unaided by the scalpel, to penetrate the veil which has so long obscured the operations of the nervous system ; yet it cannot escape the recollection how many theories, none, perhaps, so important, but bearing equally with this the semblance of probability, have ultimately been classed in the number of ingenious but unfounded speculations. Such being the lesson that experience has taught us, it is, perhaps, not too much to assert, that if the physiologist be anxious to establish, by the results of his inquiries, a great principle of the animal economy, he must be satisfied previously to submit his conclusions to the test of anatomy."

It may perhaps be considered a strong mode of expressing himself, when Mr. Grainger tells us that our common physiological opinions are absolutely opposed to the dictates of common sense. But we shall see presently, whether that case is established.

The work itself consists of seven chapters and an appendix. The first chapter contains—A Brief Review of the Opinions which have been entertained, relative to the Functions of the Spinal Cord;—the second chapter—The Properties of the Grey and Fibrous Substances;—the third chapter—Anatomy of the Spinal Cord and Nerves;—the fourth chapter—Physiology of the Spinal Cord;—the fifth chapter—General Results;—the sixth chapter—Theory of the Functions of the Sympathetic Nerve;—and the seventh—Theory of Muscular Action.

### I. A brief Review of the Opinions which have been entertained, relative to the Functions of the Spinal Cord.

We may readily admit that, great as have been the modern discoveries of Bell, Mayo, Magendie, and others, in reference to the functions of the nervous system, those functions are still imperfectly understood, and that our ideas of it are vague in many instances, and contradictory in some.

The questions that more especially affect us at present are the following:

1. What is the seat of sensation and volition?

2. What are the powers and attributes of the spinal cord?

3. Is there any mechanism by which those properties of the spinal cord and medulla oblongata, which have been more particularly explained by Dr. Hall, and are now known as the "excito-motory," are effected?

1. The opinions on the precise seat of sensation and volition are unsatisfactory and conflicting. Some have attributed these faculties to the spinal cord—the majority have placed them in the medulla oblongata—some have considered them the property of the cerebral hemispheres. Much of the confusion has resulted from not distinguishing with accuracy the "excito-motory" actions. Mr. Grainger's sentiments are expressed in a passage which proclaims him one of the class of physiologists who regard the cerebral hemispheres as the sole depositories of these important properties.

"All these details sufficiently prove that the opinions which prevail respecting the seat of sensation and volition, are anything but satisfactory. Although it is with great diffidence that I venture to disagree from so many high authorities, yet, I cannot refrain from expressing my conviction that those physiologists who contend that sensation and volition are properties either of the spinal cord in general, or of that part of it more particularly which is placed in the cranium, are equally in error. I believe it is susceptible of proof, although this has not yet been satisfactorily accomplished, that the brain is the sole organ of sensation and volition; and that the spinal cord is only connected with the production of true voluntary motion, in consequence of one part of its structure serving as the conductor of the volitions of the cerebrum." 11.

2. Setting aside the possession of sensation and volition, of which we have, at present, deprived the spinal cord, it is universally admitted to serve as the conductor of these faculties, and it is now generally admitted also, to possess with its nerves the "excito-motory" function.

"It is certain," observes Mr. Grainger, "that the spinal cord has a most important and immediate connexion, with the arteries of the muscles which are called voluntary; that it enjoys a power of exciting these to contract independently of the brain; and that it is this circumstance which has been the cause of all the conflicting opinions and evidence, which have been advanced on this subject. The discovery by Dr. M. Hall and Professor Müller, of the real nature of the reflex function of the spinal cord, appears to afford a clue to the whole of this mystery; and when developed to its full extent, is probably capable of explaining most, if not all, of those anomalies which so seriously obstruct the successful cultivation of the physiology of the nervous system.

It was reserved for Dr. M. Hall to penetrate the mystery which had baffled all other physiologists; and to prove, not only that the phenomena which result from the reflex action of the spinal cord are essentially distinct from sensation and volition; but, likewise, to perceive, what had never before been surmised, the necessity of an independent division of the nervous system, equally distinct from the great sympathetic and the true cerebral system, by the agency of which these peculiar phenomena are accomplished. Until these important distinctions were announced, no physiologist could explain how those motions, which are usually termed involuntary, but which must now be called excited, could take place in muscles of a voluntary character. How, for example, the actions of the diaphragm, which are susceptible of being suspended and otherwise controlled by the will, continue nevertheless during sleep, in coma, in the anencephalous infant, and in animals experimentally deprived of the brain; how the muscles of the face, which are so immediately under the influence of volition, become excited, together with a multitude of other muscles, in sneezing: how the muscles of the throat, which in speaking and singing are so obedient to the mental impulse, are placed beyond its control in swallowing, coughing, and vomiting: or, in fine, how the muscles of volition can be excited to contract by impressions made on the sentient surface of the skin, when all volition and sensation are destroyed by the section of the spinal cord. These, and a multitude of other apparently conflicting phenomena of the nervous system, may be all readily solved according to the views of Dr. M. Hall." 13.

We need not go into the hypothesis of Dr. Hall, having lately devoted an article to its exposition. But this we will say. We were at first inclined to think Dr. Hall's opinions fanciful, and to constitute no great improvement on previous knowledge. But time and reflection, and the development on Dr. Hall's part of his own views, have led us to attach a much higher degree of merit and importance to them. We have observed, at the same time, with little surprise but with less satisfaction, the attempts of several persons to foist on the public some obscure hints, and some mystical conjectures as complete anticipations of the elaborate generalizations of Dr. Hall. This is equally unjust and foolish, and will injure that gentleman as little as it will benefit those who advance such claims.

3. In answer to the third question, we may observe that, hitherto, no mechanism has been discovered, for the express performance of the duties of the excito-motory system. But it is Mr. Grainger's object to ascertain whether any such mechanism exists.

On the whole, we may sum up, in the words of Mr. Grainger, that notwithstanding certain difficulties and anomalies that never have been explained, the prevailing opinion has been, and still continues to be, that sensation and volition are properties of the medulla oblongata, and consequently that they remain after the loss of the brain.

It is further evident, that many writers suppose the spinal cord also to be more or less connected with sensation, and especially with the production of voluntary motion. But although these doctrines are so generally received, there are few physiologists, who do not perceive that they are utterly insufficient to explain the phenomena of the nervous system, and that they are daily contradicted by the effects of disease.

### II. Properties of the Grey and Fibrous Substances.

Of course it is a great object to ascertain the precise properties of the grey and fibrous substances found in the central organs of the nervous system. But the inquiry is attended with difficulty, and our knowledge will be found to be rather inferential and analogical than direct and satisfactory.

1. To Gall and Spurzheim are due the merits of having called public attention to the anatomy of the nervous system, and of having originated the great discoveries of our days which hinge essentially upon that anatomy.

Gall and Spurzheim contended that the grey matter secretes or forms the white fibres, and that this is its principal office.

This opinion is contradicted—first, by the observation of Tiedemann, that the fibrous matter appears before the grey; secondly, by the fact, that the material of the nervous system is derived from the plastic substance, or blastema of the embryo; thirdly, by all analogy.

2. The doctrine most commonly received at the present day considers the fibres of the white matter as subordinate to the grey.

The main reasonings on which this opinion is founded are the following:—1, The importance of an organ in the economy is dependent on and may be measured by the quantity of blood that it receives. Now the grey nervous matter is demonstrably much more vascular than the white. 2, The grey matter is found to increase in quantity in the ratio of the nervous energy. The observations of Mr. Grainger upon this head put the case so well, and are so adequate for the purpose, that we cannot do better than transcribe them.

"A second circumstance bearing upon the present question is, that *the grey matter increases in quantity in the exact ratio of the nervous energy.* We learn from a comparative examination of the brain, that the intellectual operations become diversified and energetic in proportion as the grey substance is accumulated; and it is in this respect especially, more than in that of relative volume, that the brains of the lower animals differ when compared with each other, or with the human cerebrum, the great peculiarity of which consists of the very large proportion of its grey matter, when contrasted with the nerves attached to its base. A very accurate test of the intelligence possessed by different animals, and even by different individuals of the human species,* is thus afforded by the

* "In advancing this opinion, which has been supported by so many distinguished physiologists, I beg to express my dissent from the conclusion attempted to be drawn from it by some writers, that such a theory displays the character of materialism. The merits of that long-disputed question are not touched by the observations offered in the text; for they merely relate to the development of the convolutions, or, in other words, of the grey substance; for the so-called convolutions of the brain are only another illustration of that principle so beautifully displayed in the formation of the glands, according to which the largest possible quantity of material is contained in the smallest possible space.

But the condition of the cerebro-spinal axis, at the time of birth, affords, perhaps, the most satisfactory evidence on this point. At that period, the grey matter of the cerebrum is well known to be very defective, so much so, indeed, that the convolutions are as it were in the first stage of their formation, being only marked out by superficial fissures, almost confined to the surface of the brain: whilst at this identical period, the spinal cord, owing to the imperfect development of its fibrous part, (which, as will be subsequently shown, is allied with the exercise of sensation and volition,) contains a larger quantity proportionably, of grey matter than it does in the adult; in consequence of which, according to the remark of Professor Arnold, that matter, which in the adult is placed so deeply in the interior, approaches much nearer to the external surface. Now at this particular time, the true cerebral functions, consisting of the intellectual faculties, sensation and volition, are almost entirely, if not for a brief period totally wanting; whilst the true spinal functions are in full activity. It is impossible to adduce any more striking proof than this, to demonstrate that the extent of the power inherent in the nervous system, depends on the quantity of the grey matter.

Professor Tiedemann, in his valuable work on the development of the brain, has incidentally mentioned a fact which bears on this inquiry: he has found that in the torpedo, there is a mass of grey substance placed in connexion with the fifth and eighth nerves supplying the electrical organs, larger in size than the cerebellum itself, whilst in the common skaite no such mass exists. An exactly analogous fact is furnished by the comparative anatomy of the lobe of the olfactory nerve; for, in animals distinguished by the acuteness of their smell, that body is remarkably large when contrasted with those in which that sense is less perfect. The object of such formations cannot be mistaken; it is evidently to generate power.

Lastly, it may be mentioned in corroboration of the opinion here advanced, that the grey matter is only met with in those parts of the nervous system which are known to be the seat of power; that is to say, in the encephalon, the spinal cord, and the ganglions; it is wanting, notwithstanding the assertion of Monro to the contrary, in those parts—namely, the nerves—which are proved not to have the capability of originating power." 21.

3. On a careful consideration of the preceding facts, and of the conclusions towards which they point, it will appear that the most important offices of the nervous system are in all probability performed by the grey matter. If this be so, the part played by the white must be subordinate. Conduction of the nervous power is seemingly its office. In the nerves such conduction is evident enough, as well as the incapability of originating power. In the brain the proof of this becomes more difficult, the white fibres and grey substance being so inseparably mingled. But the dissections of the hardened brain corroborate, to a great extent, the suggestions of analogy, and point

---

material instrument by which the nervous power, whatever it may be, operates. Even the inquiry respecting the character of that power, which I consider to be a most legitimate subject of physiological inquiry, has nothing to do with the nature of the soul; the two questions are essentially distinct from each other, and as such they ought to be treated."

out, in apparently a satisfactory manner, fibres of sensation and volition proceeding from the spinal cord, transverse and longitudinal commissural fibres, and peripheral fibres uniting the individual convolutions. Microscopical researches would seem to discover "*varicose fibres*," peculiar to the white substance of the cerebrum; but the microscope has played us false in anatomy too often to challenge much respect from us. We may conclude then that, in the present state of our knowledge, it is most reasonable to believe that the phenomena exhibited by the cerebro-spinal system are dependent on the grey matter, and that the white is the medium of conduction. Whether this is the whole truth, it would be folly to affect to determine.

### III. Anatomy of the Spinal Cord and Nerves.

It is not necessary to enter fully into the contents of this chapter. Many of the facts alluded to in it are more or less familiarly known; and we shall principally confine ourselves to what appear to be novel statements on the part of Mr. Grainger.

1. The *grey matter* of the cord consists of two crescentic portions, one in each lateral segment of the cord, so disposed that the concavity of either crescent looks outwards. These two portions are invariably connected by an intermediate band of grey matter, which stretches across the median plane, just behind the bottom of the anterior median fissure of the cord. Thus, the two sides of the cord are in communication with each other.

2. By a longitudinal section, it is seen that the grey matter passes continuously from below upwards. Although it varies much in colour, and is more or less intermixed with fibrous substance, it may be observed to pass continuously through the medulla oblongata, the pons Varolii, the optic thalami, and the striated bodies, on the outer border of which it is known to cease; never in this direction becoming continuous with the grey matter of the convolutions. The grey matter of the crus cerebri towards the inner side, is also joined with that of the pons Tarini, tuber cinereum, locus perforatus anticus, and thus with the neighbouring convolutions of the anterior and middle lobes of the brain. Although there is no direct junction between the grey matter of the cord and that of the optic tubercles, yet as these bodies are united with the optic thalami by grey substance, they are thus also brought into connexion with the cord itself. In the same way as the influence of an impression is transmitted from one side of the cord to the other, by the transverse process of grey matter, it is probable that by these connexions in a longitudinal direction, impressions, if sufficiently intense, are so transmitted that all the muscles may be stimulated, as happens in violent coughing and sneezing, and even probably in traumatic tetanus, from the head to the foot.

3. The relative proportion of grey matter is much greater in young than in adult animals. Exactly the reverse obtains in the cerebrum, the convolutions of which, in very early life, are exceedingly imperfect.

4. The *fibrous matter* displays on the external surface of the cord certain fissures, dividing it into a number of columns. Of these fissures, two are placed on the median plane, one on the anterior, and the other on the posterior part; besides these, there are on each side two others, the anterior and posterior lateral. The median fissures, of which the anterior is much the deepest, are for the reception of processes of the pia mater, which not only serve to convey blood-vessels towards the interior, but likewise to support and steady the divisions or columns of the cord; the anterior and posterior lateral fissures also give entrance to blood-vessels, but in addition they transmit certain filaments of the spinal nerves, which in these situations dip inwards towards the central part of the cord.

The columns thus formed are seen, on examination, to be composed of longitudinal fibres.

"It has been very generally admitted that of these columns, the anterior is for the transmission of volition, and the posterior of sensation, whilst the middle has been supposed by Sir C. Bell and others to be in a more especial manner connected with the movements of respiration. But it is very doubtful if all this be correct; for though the spinal cord undoubtedly contains a number of fibres anatomically and physiologically distinct from each other, for the transmission of volition and sensation, yet it is by no means certain that these are placed in the anterior and posterior columns respectively. On the contrary, Sir C. Bell has himself stated, that he has not hitherto succeeded in tracing a connexion between the posterior or sensitive roots of the nerves and the posterior column; and Mr. Swan remarks that there is a direct line of separation between the anterior and posterior columns placed next the median plane, and that lateral part which gives origin to the nerves. My experience entirely coincides with this latter statement; as, notwithstanding the most careful examination, I have never been able to trace any fibres from the nerves into the fasciculi composing the anterior and posterior columns; the anterior and posterior lateral fissures appear definitely to limit the two roots. As the properties of these columns will be again noticed in a future chapter, it is only necessary to remark further, that their fibres approaching the medulla oblongata, form a distinct decussation not only from side to side, as is seen in the corpora pyramidalia, and in the cords lately described by Sir C. Bell, but also as Mr. Solly has shown from before, backwards; that after this remarkable disposition, the fibres are still continued uninterruptedly upwards, till they reach the convolutions of the cerebrum and the laminæ of the cerebellum, where they terminate, after presenting a connexion with the grey matter, somewhat similar to the incrustation of the white fibres in the corpus striatum." 31.

On opening the anterior median furrow and separating the two lateral columns, a white layer is perceived at the bottom, the direction of the fibres of which it is difficult to determine; a somewhat similar structure is seen in the posterior median fissure. Gall and Spurzheim have represented a commissure consisting of transverse fibres joining the lateral columns; and Mr. Swan speaks of transverse threads passing from one side to the other. These fibres Mr. Grainger has satisfactorily distinguished.

5. *Anatomy of the Spinal Nerves.*—We need scarcely observe, that each spinal nerve has two roots, each of which apparently terminates in the lateral fibrous columns above described. Attempts have been made to trace these origins deeper, and Mr. Grainger gives the following account of them.

"Although Vicq. d'Azyr had formed a similar conjecture, it appears that Gall was the first anatomist who distinctly announced that the spinal nerves are connected with the grey substance of the cord. Bellingeri subsequently adopted this opinion; but he, with justice, opposed the idea of Gall, that all the fibres of the nerves are joined with the grey matter. Bellingeri attributes, both to the anterior and posterior roots, a triple origin; the former arising by two of its roots from the white fibrous parts, and by the third root, *perhaps*, from the grey matter; the latter arising by two roots, from the fibrous part, and by the third from the posterior horn of the grey substance. Mr. Mayo, who in one place distinctly affirms that the origin of a nerve is always in part from grey matter, speaks more doubtfully of the double connexion with the grey and fibrous substances; for, when treating of the spinal nerve, he says, that the filaments of the two roots *appear* to be partly continuous with the white fasciculi of the cord, and partly to originate in the interior of the grey matter. In an earlier edition of his Physiology, Mr. Mayo has given a representation of this two-fold connexion, which is, however, very incorrect, and has been omitted in the later editions of that work.

Keuffel, Ollivier, and Weber likewise state that the nervous fibres may be followed into the grey substance, a connexion which is also admitted by Professor Arnold.

Several distinguished anatomists deny, however, the possibility of demonstrating the union of the fibres of the nerves with the grey substance of the cord. Sir C. Bell, in allusion to this subject, says, 'Some authors describe these roots as derived from the cineritious matter; this is quite at variance with my dissections.' Bellingeri himself is not certain that the anterior root is thus connected; whilst Desmoulins denies the connexion altogether." 33.

Desmoulins, indeed, contends that the grey matter does not exist in the spinal cord of reptiles and fishes, but that there is in the centre a canal filled with a serous fluid; but, as Mr. Grainger observes, there are many reasons for hesitating before we subscribe to this assertion.

Rolando describes the nerves as being connected only with the white substance. Professor Müller gives no positive opinion. Mr. Swan thinks it doubtful whether the nerves are connected with the grey matter of the cord. Mr. Grainger has examined the subject with care, and appears to have ascertained some important facts. It is impossible in articles of this nature, to abbreviate description materially. Selection is more necessary, or more practicable than analysis.

"In considering," says he, "the interesting phenomena related by Dr. M. Hall it occurred to me, that it might be possible to demonstrate the separate existence of what he has called the incident and reflex fibres; and I was thence induced to dissect, with much care, the two roots of the spinal nerves. After repeated examinations, I satisfied myself that each was connected both with the external fibrous part of the cord, and the internal grey substance. The following is what appears to be the structure: after the two roots have perforated the theca vertebralis, and so reached the surface of the cord, it is well known that their fibres begin to separate from each other; of these fibres some are lost in the white substance, whilst others, entering more deeply into the lateral furrows, are found to continue their course, nearly in a right angle with the spinal cord itself, as far as the grey substance in which they are lost. But this arrangement has no resemblance to the distinct division into fasciculi depicted by Mr. Mayo; on the contrary, it is with great care only that small, delicate, and individual threads or striæ, as it were, are traced, dipping into the lateral fissure, and at length joining the grey matter. This difficulty is owing to the fact, that whilst the fibres on the outer surface of the pia mater adhere very intimately with that strong membrane, on its inner surface, the neurilema becomes so extremely delicate, that the fibres lose much of their firmness, and break on the application of the least force; an accident which always happens, if the pia mater be raised from the surface of the spinal cord, beyond the point where the nerves are attached. When the filaments have penetrated into the fissure, they lose their rounded figure and become flattened, and are then seen passing to the grey substance at a right angle to the longitudinal fibres of the cord. It is extremely difficult, owing to the delicacy of the parts, to determine the exact relations which exist between the above filaments and the grey matter ; but in a few dissections, I have been able to perceive these fibrils running like delicate striæ in the grey substance. In one instance the fibres being more distinct than usual, an appearance was presented having a remarkable resemblance to that which is seen, on making a section of the corpus striatum in a recent brain, after the method of Spurzheim. My friend and colleague Mr. Cooper, in this case counted distinctly five separate fibrils passing from the anterior root of one nerve, and there were some other fibres derived from the same root, which were not so plainly seen.

From numerous examinations I am induced to believe, that whenever the white fibres of the nervous system become connected with the grey substance, whether in the different masses of the brain, in the spinal cord, or in the ganglions, the arrangement is similar to what is seen in the section of the corpus striatum, to which reference has just been made. The fibres become as it were encrusted with the grey matter, a disposition which may even be seen by a careful inspection in the convolutions of the cerebrum, in which the radiating fibres of the crus cerebri are observed like delicate striæ.

In examining the roots of the nerves I have always relied on the assistance of the naked eye only, avoiding, for fear of deception, the use of a lens ; it also appeared to be preferable to dissect the parts quite in their recent state, so that the natural structure was entirely preserved. The method of Reil which is so useful in tracing the fibres of the brain, is quite inapplicable in the present case ; and Bellingeri has shown that the use of acid renders it very difficult, to distinguish the nervous filaments from the blood-vessels." 36.

Mr. Grainger had demonstrated this arrangement to his friends, some months before he visited Germany. There he met with so much disbelief, that he again demonstrated it to the entire satisfaction of Professor Bischoff, of Heidelberg. Mr. Grainger is convinced that only a part of the fibres belonging to the two roots are attached to the grey substance, a considerable number of threads being lost in the fibrous part of the cord. Many speculations have been hazarded with respect to the exact mode of communication; but, in point of fact, we know nothing positive concerning it.

The cranial nerves resemble those of the vertebral canal, in being attached both to the grey and the fibrous substances. On this point Mr. Grainger dwells at some length, and as it is an important one, we shall follow him into details.

A. *The Third, or Motor Oculi.* It is stated by Mr. Mayo, that this nerve arises by many fibrils, from the black matter in the crus cerebri. This account is perfectly correct, as far as it extends, but it is not complete ; for, as Mr. Solly has shown, some of the fibres are attached to the motor cord in the pons Varolii. But what is particularly interesting is, that after the fibres have spread out into the grey matter, or locus niger, some of them may, with care, be followed into the fibres which constitute the upper portion of the crus. Now, this part of the crus cerebri has been described by Sir C. Bell, as receiving certain fasciculi derived from the posterior or sentient column of the spinal cord, where it forms the calamus scriptorius of the fourth ventricle, and which subsequently pass upwards, through the thalami nervorum opticorum, to the cerebral hemispheres. In this manner, as the optic nerve in its origin is united with this tract, an intimate relation is established between it and the motor oculi; which, Mr. Grainger conceives, is connected with the interesting phenomenon noticed by Mr. Mayo, that, on pinching the divided cerebral end of the optic nerve, the iris contracts.

B. "In considering the disposition of the incident and reflex filaments of the posterior and anterior roots of the spinal nerves, it occurred to me, that, after being incrusted as it were by the grey matter, like the fibrils of the portio major of the fifth, in their course through the Gasserian ganglion, these two orders of fibres might become continuous with each other, and thus offer a satisfactory explanation of the mode in which impressions made on the nerves of the skin, are transmitted to those of the muscles; and although, owing to the extreme delicacy of the structure, I have not succeeded in tracing this connexion, the analogy of the third nerve renders it very probable that such an arrangement of the nervous fibres does in reality exist." 40.

C. *The Sixth, or Abductor Nerve,* proceeds our author, is usually described as arising from the corpus pyramidale; but, if the longitudinal fibres of that body be cautiously separated with the point of a fine instrument (a cataract needle, for instance), it will be found that the larger part of the nerve sinks deeply into the substance of the medulla oblongata, and there, assuming the form of a flat cord, at length reaches the grey matter. It is difficult to perceive with what fibres of the cord this nerve is connected.

D. *The Facial Nerve,* which, at the lower edge of the pons Varolii, closely approaches the descending trunk of the portio major of the trigeminal, seems to be joined, by a few fibres, to the grey matter in the interior of the pons, and upper part of the medulla oblongata.

E. *The Sublingual Nerve,* by raising the pia mater as far as the outer border of the pyramidal body, may be observed to send a few fibres into the grey substance placed on the inner side of the corpus olivare.

F. "*The Portio Major of the Fifth,* and the pneumo-gastric, in consequence of the very important part they bear in the excito-motory phenomena, require to be especially considered. The former passes, as it is known, through the whole substance of the pons Varolii, and ultimately reaches the posterior column of the medulla oblongata. In this situation, closely approaching the origins of the auditory, facial, glosso-pharyngeal, and pneumo-gastric nerves, it becomes attached not only to the white fibres, but also to a mass of grey matter, apparently continuous with that which is placed in the floor of the fourth ventricle. The remark of Mr. Mayo, that this portion of the fifth and the portio dura rise together, is full of physiological interest." 41.

G. *The Glosso-pharyngeal and Vagus.* Some of their fibres, after passing very deeply through the ascending fasciculi of the corpus restiforme, reach the grey matter placed in the posterior part of the medulla oblongata; the connexion with the fibrous structure is not so evident. Mr. Solly considers the corpus olivare to be the ganglion proper to the pneumo-gastric nerve; but no fibres of the nerve can be traced into the grey matter of that body, in the human brain. Two roots of the vagus have been spoken of, but it is difficult to demonstrate them; nor, although the two portions of the glossopharyngeal may be seen in the foramen lacerum posterius, where that nerve presents the ganglion discovered by Müller, has Mr. Grainger been able to trace them as separate roots into the medulla oblongata. Yet, as he observes, the probabilities are greatly in favour of the opinion that two roots do exist.

H. Mr. Grainger next dwells on the comparative anatomy of the cord. But we are unable to follow him.

I. On the whole, the investigations of Mr. Grainger go towards the confirmation of the opinion which has latterly been all but universal:—that, the spinal cord is not merely an appendage of the brain. Its two substances are in all probability endowed with distinct powers, and, probably also, the grey matter in the cord has its peculiar powers. Mr. Grainger surmises, that—

"In fact, it constitutes, with the nervous fibres attached to it, the true spinal cord, the existence of which, as a structure independent of the brain, was first declared by Dr. M. Hall.

The second portion of the spinal cord consists of the white fibres, all of which, after a most complicated disposition, seem to extend to the convolutions of the cerebrum and the layers of the cerebellum; it may, therefore, with propriety be called the cerebral part of the cord.

The result of dissection further shows, that in the skin and all other sensitive surfaces to which the so called sentient nerves are distributed, there are, in reality, two orders of fibres essentially distinct from each other; one set terminating in the grey substance of the spinal cord, and the other in the white or cerebral fibres. In the same manner, with respect to the nerves distributed to the muscles, it is proved that each contains one class of fibres running into the grey matter of the cord, and another order ending in its cerebral portion.*

Thus, in the compound nerves of the body, there are in reality four instead of two different classes of fibres; and, when the physiology of these parts is considered, it will be made apparent, that these several fibres transmit different impressions: that of those going to the brain, the fibres derived from the sentient nerves, transmit impressions which excite sensation, and those belonging to the motor nerves, volition; that of the fibres attached to the grey matter of the cord, those derived from the sentient nerves, transmit impressions made on the skin to the true spinal cord, the peculiar power of which they excite; whilst those derived from the motor nerves, transmit to the muscles the effects of the power thus excited. Of these four classes, I conclude, that those attached to the grey matter are the incident and reflex nerves of Dr. Hall; and that they, together with that matter, constitute the true spinal or excito-motory system." 47.

It is difficult to perceive how Mr. Grainger has proved, or proves—that, "the result of dissection further *shews*, that in the skin and all other sensitive surfaces to which the so-called sentient nerves are distributed, there are, in reality, two orders of fibres, essentially distinct from each other; one set

* "It is no argument against this statement, that these different orders of fibres cannot be demonstrated in the nervous cords going to the skin and the muscles. The same kind of objection applies to the existence of the sentient and motor fibrils in the compound nerves, which cannot be distinguished mechanically from each other; and yet no one doubts the justice of Sir C. Bell's conclusion as to their independence both in an anatomical and physiological point of view."

terminating in the grey substance of the spinal cord, and the other in the white or cerebral fibres." Nor is it easy to understand how he reconciles this affirmation in his text with the contrary negation in his note;—that, "these different orders of fibres cannot be demonstrated in the nervous cords going to the skin and to the muscles."

The comparison between these fibres, which can and cannot be observed, and the two roots of the spinal nerves is imposing, but, perhaps, not perfectly fair. Those roots are obvious to the naked eye, can be handled, dissected, separately cut in living animals. Their palpable character prevents anatomical dispute—their positive separation has facilitated physiological experiment—and the direct results of the one and of the other have established the undeniable fact of the different properties of the two roots.

But the anatomical basis of the "reflex" theory, however plausible, however pretty, and however *probable*, is by no means so obvious nor so satisfactory. The different fibres, which it is necessary to distinguish, have been *seen*, it is true, by Mr. Grainger, and are admitted by Professor Bischoff; but they were seen by the one with trouble, and have been admitted by the other with difficulty, nor have the mass of anatomists yet examined or assented to the grounds of belief in either. To compare such fibres to the two roots of the spinal nerves is to compare a small quantity with a great, an uncertainty with a demonstration; and such comparison cannot, in an exact science like anatomy, be unchallenged.

So far as we can see our way, it appears to us that these investigations of Mr. Grainger's have great merit, and that they demand on the part of anatomists much attention, and an impartial scrutiny. Yet it may be said that, so far as physical discovery is concerned, he has *seen* what others have *said* they saw, or have surmised; and it is rather in the elaborate character of his inferences, and in his application of his facts to the support of the "reflex" theory, than in new views or in new facts, that he differs from his predecessors or contemporaries. But to revert to the text of that gentleman.

Having decided that there exist, besides the nerves of sensation and volition, two other classes which are anatomically and physiologically distinct, it becomes, of course, necessary for him to distinguish these different orders by separate names.

"Dr. M. Hall has proposed to call the fibres, which pass from the skin to the true spinal cord, the *incident nerves*, and those, which proceed from the true spinal cord to the muscles, the *reflex nerves*. These terms are very expressive of the powers, which these filaments probably possess of transmitting impressions in the directions indicated by their names; and, therefore, may be with propriety adopted in treatises in which the excito-motory phenomena are considered. But, as at the present time, doubts still exist with respect to the exact mechanism by which the reflex action of the cord is effected, it is advisable to select, for general purposes, terms merely expressive of the facts which are established by the evidence of anatomy, and which do not involve any hypothetical doctrine. In this manner the nerves of the cerebro-spinal axis may be divided, as, indeed, Dr. Hall has proposed, into the true cerebral, comprising the true sentient and the true volition fibres; and the true spinal, consisting of those fibres derived from the anterior and posterior roots which enter the grey matter of the cord. With these fatcts before us, the dispute, respecting the existence of what are called the cerebral nerves, is readily determined. It is well known, that whilst such nerves are admitted by some physiologists, the majority of writers in the present day contend, that all the nerves contained within the cranium, are spinal nerves. Neither of these opinions is correct; for each of the cranial nerves is, in reality, composed like those attached to the vertebral part of the spinal cord, of a true spinal and a true cerebral portion; the former being attached to the prolongation of the grey matter of the cord, and the latter, to the sensiferous and volition fibres, which ascend to the grey matter of the cerebral convolutions. The only nerve which, perhaps, consists of cerebral fibrils alone, is the olfactory." 48.

Mr. Grainger continues:—

"It is necessary, in conclusion, to point out a very important principle, in accordance with which the origin of the incident and reflex nerves is governed. From the office which has been assigned to the former, of transmitting the impressions of physical agents to the latter, it is evident that there must be, as Professor Müller has remarked, a ready means of communication between them. In obedience to this necessity, it is found that the central extremities of the incident nerves do, in reality, very closely approach the central extremities of those reflex nerves, with the function of which they are associated. This principle is very evident in all the spinal nerves, the incident and reflex fibres of which are attached to corresponding segments of the grey substance; and the same disposition is evinced in the incident and reflex fibres of the several cranial nerves, when they are traced with sufficient minuteness." 48.

Mr. Grainger alludes to Mr. Mayo's remark, that—nerves of motion take their rise from the same region or segment with those sentient nerves which transmit the impressions by which their action is usually regulated. This principle, Mr. Grainger observes, does not apply to the *sentient*, but to the *incident* nerves; for the true sensiferous or cerebral fibres are not thus attached with the volition fibres of the motor nerve, to corresponding masses of the grey matter; on the contrary, the sensiferous fibres of the posterior roots, although for the convenience of arrangement they approach the volition fibres of the anterior roots in the spinal cord, yet they continue upwards till they reach the various convolutions of the brain. Our author adds that —the explanation now offered of this fact affords a satisfactory elucidation of a principle which, otherwise, could not be comprehended; for, whatever assertions there may be to the contrary, it is certain that there is no necessary relation either between muscular contractility in general and sensibility, or between this latter property and the contraction of that portion of the muscular system, which is under the influence of volition.

### IV. Physiology of the Spinal Cord.

This constitutes a long chapter—one of 66 pages. Our space is too limited to permit us to give an accurate or an ample account of the whole. The object of Mr. Grainger is to investigate, more minutely than he has already done, the functions of the various portions of the cord. But this has formed lately so much a subject of inquiry, and has been noticed in such various ways in this Journal, that we need only select particular passages and observations.

A. *Of the Vertebral Portion of the Spinal Cord.*—We will not go over ground already trod. It is enough to observe, that there seems now good reason to believe that the cord *conducts* sensation and volition, and *originates* the reflex action, or rather forms with the incident and reflex nervous fibres the apparatus of that action. All this has been not only rendered probable, but demonstrably proved in all its parts, by reasoning and by experiment. Unfortunately, as the nervous fibres of the reflex action are not distinguishable from the nervous fibres of sensation and volition, it is impossible to divide one without the other, and so perform an experimentum crucis. The next best substitute for such an experiment is to cut across the entire cord. We thus deprive the lower portion of the power of conduction to and from the brain; but we leave the reflex apparatus. It was by an experiment of this sort that the reflex theory was first set upon its legs.

Mr. Grainger relates several additional experiments, all confirming the original one, but not necessary for its perfect comprehension. But the following observations are of a more interesting character.

"Dr. Hall has pointed out the extraordinary susceptibility of the verge of the anus, in producing the reflex action of the cord; and I have myself remarked the same thing in every species of animal—in mammalia,—in amphibia, and in insects. In connexion with this fact it is important to describe a phenomenon, which was noticed by Professor Bischoff in the green frog (*Rana arborea*) so common in many parts of Germany. Upon irritating the cloaca in one of these animals which had been decapitated, the most violent motions were excited in the hind legs, and *repeated attempts were made by these limbs to remove the instrument*, with which the cloaca was touched. This fact I have since repeatedly seen, in the green and common frog, both when the head was removed* and when the spinal cord was divided in the back; the same thing may readily be noticed in the common fly and other insects, after decapitation. I have also observed that if, after having cut off the head in frogs, fire is applied to the fore part of the trunk, violent motions to remove the source of excitement are made. It is impossible not to be struck with the analogy of this curious phenomenon, occurring, it must be remembered, when it can be proved that sensation and volition were destroyed, and the experiments of Magendie; in which, after the removal of the cerebrum and cerebellum, the animal employed its feet to remove a source of irritation from the face, caused either by plucking a hair of the whisker, or by dropping concentrated acid on the nose."† 60.

Mr. Grainger proceeds to shew, that, as recent observations have established the analogy between the nervous system of the articulata and the cerebro-spinal system of the vertebrata, the experiments on the excito-motory actions in the former acquire an increased interest. Those experiments do not require specification, the general results being sufficient for our readers. Those results are briefly summed up by Mr. Grainger. They are found, when carefully considered, to correspond in character with those obtained by vivisections practised on the vertebrata; the only difference relating to the extent of the reflex action. In both cases, there is the same

* "It is necessary in batrachian reptiles to be very careful in removing the head, that the section is made sufficiently low, or otherwise a part of the brain remains attached to the body."

† "The motions above noticed are best seen when the cord is divided high up towards the neck."

absence of all spontaneous motion, in the parts cut off from their connexion with the brain; the same convulsive motions arising from time to time, without any external irritation; the same kind of combined motion, when the surface is stimulated; and the same modification in the intensity of the excited actions, according to the part upon which the impression is made.

B. *Of the Cranial Portions of the Spinal Cord, or Medulla Oblongata.*—The functions of the medulla oblongata have been and are a subject of dispute and difficulty. The cranial portion of the cord comprises not only this, but the crura cerebri, the optic tubercles, and the fibrous, perhaps, even the grey matter of the optic thalami.

The medulla oblongata is clearly proved to be indispensable for the performance of respiration, and of two important acts, sucking and deglutition. It has been supposed to be the seat of sensation and volition.

Mr. Grainger rightly observes, that the investigation of the endowments of the medulla oblongata may be prosecuted with the greatest advantage, by considering, first, the effects of vivisections performed on the brain; secondly, the true nature of the phenomena displayed by anencephalous infants; thirdly, the evidence afforded by pathology.

We cannot enter into an investigation so elaborate. We may content ourselves with observing that Mr. Grainger decides against the medulla oblongata being the seat of sensation and volition, which he thinks, with others, reside in the cerebral lobes. He shews that the medulla is concerned, in a very important degree, in the production of excitomotory phenomena of great consequence.

Mr. Grainger's remarks on some of the recent vivisections and on Magendie, the arch-vivisector of modern times, accord so perfectly with the sentiments of the most philosophical anatomists of the day, and are so calculated to make an useful impression on the minds of young anatomists, that we cannot refrain from extracting them. Speaking of Magendie, he says.—

"I approach his labours connected with the nervous system with much regret; because I feel myself compelled to state, that, so far from admitting that they tend to remove the veil which obscures the operations of the brain, I conceive that, viewed in the aggregate, they have constituted the great barrier to the progress of modern physiology. In stating this opinion, I only give expression to a sentiment entertained by some of the most profound physiologists of Europe; who perceive that the doctrines, advocated by systematic writers, and which have mainly originated in the researches of M. Magendie, are totally insufficient to explain the phenomena of muscular action, and are, in many respects, contradictory of each other. It is acknowledged by all parties, that the results of vivisections, particularly those which relate to the mental operations of animals, in which it is so difficult justly to interpret the effects produced on their feelings, must be received with the greatest caution. There are, in fact, difficulties inseparable from such investigations, in whatever manner they may be conducted; but these difficulties are immeasureably increased when experiments are performed on living animals, not to test the correctness of opinions founded on an antecedent process of reasoning, but to open, as it were, the chapter of accidents, and to endeavour, by a lucky chance, to discover something new. In this respect, the method pursued by M. Magendie is most objectionable; as it is apparent that his mutilations were practised with no definite object, but with the design of wresting, from their results, some conclusions respecting the most mysterious phenomena of the animal frame. But nature is not thus to be forced into a disclosure of her hidden truths ; success, when it is to be obtained by experimental inquiry, which when judicially applied is a very important means of discovery, can only be commanded by making these manual operations subservient to comprehensive views of the laws, which regulate the structure and functions of organized bodies. Such being the unphilosophic spirit in which these experiments were conducted, we are justified in viewing with doubt the conclusions they are supposed to warrant ; especially when it is recollected, that, in the same work in which they are related, doubts are expressed whether the olfactory is the nerve of smell, the optic the nerve of vision, or the auditory the nerve of hearing." 68.

It is doubtful whether, as a general truth, experiments ought to be solely instituted with the view of supporting or negativing certain propositions. This is one mode and a very excellent, indeed the best, one, for advancing truth. But experiments of a less definite character have often been productive of great results, for discovery does not run in the groove of human calculation or foresight.

In the particular instance, to which Mr. Grainger's observations apply, they are probably quite unobjectionable. Experimental vivisections are so cruel, so opposed to the best feelings of our nature, that they should ever be undertaken with reluctance, and prosecuted in the most sparing manner. If a chemist consumes uselessly some bottles full of gas, the only mischief is a little loss of time and matériel. But if a dozen dogs or pigeons are physiologically tortured to death, with the vague expectation of some curious fact turning up during the process, there is a great wrong done to humanity and probably no great good to science. In the case of M. Magendie both results have taken place. His experiments, not being philosophically instituted or directed, have tended to confuse the plainest truths, and to establish the most extravagant fancies.

Mr. Grainger gives an abstract of the principal experiment of Flourens, on the consequences of removing the cerebral lobes. He shews that that experiment goes far in supporting the excito-motory hypothesis ; the phenomena exhibited by anencephalous fœtuses he analyses and explains upon the same hypothesis ; and, after detailing some experiments and reasoning and some facts, he concludes that :—they establish the position that the action of the lips in sucking, and the movements of respiration can, and do, take place in anencephalous fœtuses, in animals deprived, experimentally, of their brain, and in *all* new-born animals, without the agency of consciousness, sensation and volition.

The phenomena of *deglutition*, and of *respiration*, are considered in reference to the same hypothesis, and shewn to accord with, and support it. With respect to *respiration*, our readers may be aware that Dr. Hall considers the vagus nerve as the excitor of the respiration, in consequence of transmitting the impression made by the carbonic acid on the surface of the air cells to the medulla oblongata, by the power of which the phrenic nerve is excited, and the diaphragm made to contract. But, after detailing some experiments in imitation and in continuation of Brachet's, Mr. Grainger observes with respect to them :—

"The results of these experiments are partly corroborative of those performed by M. Brachet ; but it is certain that in the first there *was some uneasiness* produced; and yet, if the pneumo-gastric be the only conductor of impressions from the lungs to the brain and to the spinal cord, causing in the former organ sensation, as when the breath is held, and in the latter, the action of the diaphragm, it is certain that *no pain* ought to have been excited in the first experiment. But, besides this difficulty, there are others which have not been explained. Dr. Hall conceives that after the division of the vagus, an animal breathes by means of its volition ; and, as there is no doubt that the phrenic, like all the motor nerves of the spine, has two roots, one spinal and one cerebral, there is certainly nothing to prevent the act of the will on the diaphragm. If, however, it is by the will that breathing is continued in these circumstances, it is certain that there must be some previous sensation excited, that the animal must experience some painful impression, the 'besoin de respirer,' which according to M. Brachet and Dr. Hall is destroyed by the division of the vagus.* These circumstances induce me to conclude, that painful impressions *are* transmitted to the cerebrum after the section of the pneumo-gastric; and, it is probable, that this takes place through the medium of those branches of the great sympathetic, which are distributed to the lungs. It is well known, that although impressions made on parts of the body, such as the mucous surface of the intestine, which receive no other branches than those of the sympathetic, are not, under ordinary circumstances, carried to the brain ; yet, if the impressions are sufficiently intense, they are so transmitted, and pain is the result. It is thus possible to conceive that volition may be excited, and that it is in this manner the action of the diaphragm is maintained after the section of the vagus. The only objection to this supposition is, that birds, and even dogs, and other animals, have been known to survive the division of these nerves several days ; during which lengthened period, it is difficult to admit the possibility of voluntary action being continued, so incessantly, as occurs in respiration.† I mention all these circumstances, not because I doubt the correctness of Dr. Hall's theory; but to show that this is a subject requiring further investigation." 90.

We fancy that there are few points connected with the excito-motory doctrines, which do not require careful consideration and prolonged inquiry. What we are about to state will tend we think to support this suspicion.

In considering the evidence afforded by *pathology*, Mr. Grainger relates a case communicated to him by his colleague Mr. Barron, and makes some observations on it which he subsequently expands and illustrates.

*Case.* A girl, about fifteen years of age, who was a patient of Mr. Crosse at the Norfolk and Norwich Hospital a few years since, was affected with angular curvature of the spine, producing insensibility and paralysis of the lower extremities. On tickling *the soles of her feet,* which as an experiment was often done, the legs were immediately slightly retracted, although the patient said she felt nothing ; it was further remarked, that, on touching the *other parts of the feet* or *the legs* in the same manner, no effect was produced.

"The results," says Mr. Grainger, "noticed in these cases are full of interest. They prove, first, *that in parts of the body indisputably deprived of all feeling and power of voluntary motion, contractions may be excited in the so-called voluntary muscles, by impressions made on the skin;* secondly, *that this capability of exciting*

---

* "The above statement is not at all opposed to the opinion advanced in another part of this Treatise, that sensation is not a necessary antecedent to volition."

† "M. Brachet attributes the actions of the respiratory muscles, which continue after the division of the pneumo-gastric nerve, to custom or habit; but this is an untenable position ; it is certain that no muscle ever contracts without some definite cause of excitement."

*muscular contractions, is not equally possessed by all parts of the external surface of the body; but that the sole of the foot, which in walking comes in contact with the ground, is that precise part in which the action is excited in the most energetic manner.*" 95.

We pass over some pages to Mr. Grainger's inquiry into the causes of the *motions of the limbs.*

That these are voluntary every body knows. Dr. Hall thinks that to the excito-motory system is owing their *tone.* But Mr. Grainger advances a much more bold, if not a startling, proposition. After criticising some doctrines and adverting to some experiments, he proceeds to remark:—

"These are striking facts, and indicate the existence of a power, by the operation of which the motions of progression may be excited, when volition is destroyed. So deeply impressed is the mind, however, with the idea of our motions in walking being altogether voluntary, that it is almost impossible to conceive how the mere circumstances of the foot coming in contact with the ground can cause the muscles of the lower extremities to produce all the complicated actions, which are necessary to take another step; and yet it is certain that these motions are regularly effected, when the mind is entirely abstracted from all thoughts of the actions that are required; so that at these times, which are so extremely frequent, the movements of progression become automatic. It is often said, indeed, that under such circumstances, the muscles contract from habit; but such a vague explanation becomes quite insufficient, when it is recollected how invariably every natural phenomenon is preceded by a definite cause." 115.

It must be admitted that a position of such a description as the preceding requires some examination. Mr. Grainger anticipates several objections to it. It is necessary for him to call in the aid of volition, or an antagonist to the excito-motory actions. It is certain, he says, that volition not only possesses the power of exciting what are called the voluntary muscles, but that it can also so control them, as to stop the effect of the impressions transmitted by the incident nerves. The case of the diaphragm is one in point.

"If the excito-motory phenomena be carefully investigated, it becomes evident that this controlling influence of the will is regulated, according to the wants of the economy, in obedience to certain laws. Thus those motions, which are of a preservative character, such as the closure of the eyelids, on the cornea or cilia being touched, or the protection of the larynx during deglutition, are altogether removed from the influence of volition; whilst, on the contrary, the muscles, which are necessary to locomotion, are, in the normal state of the system, in every animal possessing a brain, placed under the full and perfect control of the will. But, if the incident nerves are stimulated in excess, as in burns or tetanus, then no check, which the individual can exert, is able to prevent the most violent muscular contraction. Between these two extremes there are many shades; in every instance, however, the power of the will is always determined by the importance of the function, with which the motions are connected, and the intensity of the stimulus.

Lastly, it may be remarked, that, if such a modification of volition really exists, it is certain there must be conductors for this special purpose, distinct from all the other parts of the apparatus of sensation and volition. Without pretending to designate any particular structure, as being the organ of transmission, it may yet be stated that it is, doubtless, confined to the cerebro-spinal axis; in the component parts of which—the brain and spinal cord—there are various fibrous structures, such as the anterior column of the latter, the uses of which still remain to be discovered." 116.

We have discussed four of the seven chapters of which Mr. Grainger's work is composed. When we commenced the examination of the book, we did not think that it would have occupied us so long, or that so much would have become the subject of transfer to our pages. Not only, however, has there been much noticed, but more remains for notice still. We have arrived at a natural break in the subject, and we avail ourselves of it. In our next number we shall return to the excito-motory system—a system or hypothesis which must attract for some to come great attention, and which directly or indirectly will certainly revolutionize our ideas of the operations of the cerebrum and spinal cord.

We have left ourselves no space to speak of the papers in the Guy's Hospital Reports, nor of the new edition of Quain's Descriptive Anatomy, nor of Baly's Translation of Müller's Physiology. Yet they are all good in their respective ways. The papers in the Guy's Hospital Reports should be *read*—the other works should be *studied*, and to be studied they must have been *bought*. To the buying public, the students, we recommend them. Müller's Physiology is a first-rate production, and a general acquaintance with it (an acquaintance now made easy by the translation of Mr. Baly) will philosophize in a high degree the anatomists of this country. Mr. Baly deserves their thanks, and will, we are confident, meet with their encouragement.

